# Trident Sign: The Key Magnetic Resonance Imaging Finding Distinguishing Spinal Cord Sarcoidosis From Multiple Sclerosis and Seropositive Neuromyelitis Optica Spectrum Disorder

**DOI:** 10.31486/toj.24.0027

**Published:** 2024

**Authors:** Ariya Beitollahi, Hunter Berry, Paul Gulotta, Robert Morales, James Milburn

**Affiliations:** ^1^ The University of Queensland Medical School, Ochsner Clinical School, New Orleans, LA; ^2^ Department of Radiology, Ochsner Clinic Foundation, New Orleans, LA

**Keywords:** *Myelitis–transverse*, *neurology*, *neurosarcoidosis*, *radiology*, *rheumatology*, *sarcoidosis*

## Abstract

**Background:** Spinal cord sarcoidosis, an uncommon manifestation of neurosarcoidosis, presents diagnostic and therapeutic challenges because the condition is rare and has diverse clinical manifestations that can mimic other conditions such as multiple sclerosis and neuromyelitis optica spectrum disorder.

**Case Report:** A middle-aged African American female with a history of idiopathic intracranial hypertension and hydrocephalus with ventriculoperitoneal shunt presented with progressive, predominantly left-sided gait instability, weakness, and paresthesia. Cerebrospinal fluid showed lymphocytosis, red blood cells, elevated oligoclonal bands, and elevated kappa free light chains, concerning for multiple sclerosis. Neuromyelitis optica spectrum disorder testing was negative. Magnetic resonance imaging (MRI) demyelination protocol revealed normal brain imaging and a longitudinally extensive spinal cord lesion with the distinctive trident sign on contrast-enhanced axial views, consistent with spinal cord sarcoidosis. The patient was treated with intravenous methylprednisolone for 5 days, resulting in improved lower extremity strength, but ataxia and sensory deficits, especially proprioception, persisted. Follow-up imaging and examinations demonstrated continued spinal cord involvement with minimal improvement despite treatment.

**Conclusion:** Current management guidelines for spinal cord sarcoidosis are based on limited evidence, necessitating further research to establish optimal protocols. The trident sign on MRI may distinguish spinal cord sarcoidosis from conditions such as multiple sclerosis and neuromyelitis optica spectrum disorder. Early radiologic detection could improve outcomes and reduce long-term neurologic deficits. A comprehensive multidisciplinary approach is essential for effective, patient-centered care planning.

## INTRODUCTION

Sarcoidosis is a multisystem granulomatous disorder characterized by the presence of noncaseating granulomas in involved organs.^1^ While sarcoidosis frequently involves the lungs and hilar lymph nodes, 10% of patients present with extrapulmonary manifestations.^1^ The disease is most prevalent in middle-aged African American and Scandinavian females, and although the etiology remains unclear, genetic predisposition, exposure to beryllium salts, and infectious mycobacteria are believed to play a role.^2-4^

Neurologic complications affecting any portion of the central or peripheral nervous system—cranial and peripheral neuropathy, aseptic meningitis, and hypopituitarism—occur in up to 10% of patients with sarcoidosis.^1^ Central nervous system involvement most commonly involves the leptomeninges or brain parenchyma; spinal involvement is rare. Case studies of spinal cord sarcoidosis have reported central cord and dorsal subpial enhancement, described as a distinctive trident sign on axial magnetic resonance imaging (MRI) views, highlighting the diagnostic significance in identifying the condition.^5-13^

We describe the presentation, imaging features, and management of a patient with suspected spinal cord sarcoidosis.

## CASE REPORT

A middle-aged African American female with a medical history of idiopathic intracranial hypertension, hydrocephalus, and pseudotumor cerebri requiring ventriculoperitoneal shunt placement in 2015 and distal revision in 2021 presented to the emergency department (ED) with a 10-day history of progressively worsening left-sided gait instability, weakness, and paresthesia in the left lower extremity and bilateral upper extremities. She had difficulty walking and required assistive devices to ambulate. She also noted intermittent frontal headaches that improved with treatment and were not associated with visual disturbance or seizures. She denied recent trauma, loss of consciousness, fever, chills, dysuria, hematochezia, melena, hemoptysis, and hematemesis. The patient reported that she was a former tobacco smoker and smokeless tobacco user, was an active marijuana smoker, and consumed 2 to 3 glasses of wine per week.

During her initial presentation to the ED, the patient was afebrile with normal, stable vital signs. On examination, she had weakness of the left thigh and hip extensors and flexors, with intact distal reflexes. The remainder of her examination was unremarkable. Her abnormal laboratory results demonstrated hypokalemia (3.4 mmol/L, reference range, 3.5-5.1 mmol/L), hyperchloremia (111 mmol/L, reference range, 95-110 mmol/L), hypocarbia (20 mmol/L, reference range, 23-29 mmol/L), hypoalbuminemia (3.0 g/dL, reference range, 3.5-5.2 g/dL), low alkaline phosphate (49 U/L, reference range, 55-135 U/L), normocytic anemia with a low hemoglobin (9.7 g/dL, reference range, 12-16 g/dL) and low mean cell volume (81 fL, reference range, 82-98 fL), and low hematocrit (31.4%, reference range, 37.0%-48.5%). All other laboratory tests were within normal limits.

A series of imaging studies was ordered to evaluate the status of the patient's shunt and to rule out stroke. Computed tomography (CT) of the lumbar spine without contrast showed mild degenerative changes that were most pronounced at L4-L5, with mild spinal canal stenosis and mild right neural foraminal narrowing. CT of the head without contrast showed no acute findings, with stable positioning of the right ventriculostomy catheter, stable configuration of the ventricular system, and no hydrocephalus compared to prior studies. An x-ray shunt series also showed stable positioning of the ventriculostomy catheter, without kink or discontinuity.

The patient's presentation and results were discussed with the neurosurgery team, and they evaluated the patient in the ED. The team ordered a noncontrast MRI of the lumbar spine, which showed a broad-based posterior disc bulge and posterior annular fissure at L5-S1 with mild bilateral neural foraminal narrowing. After reviewing the patient's MRI, the neurosurgery team recommended outpatient follow-up with their service in 1 week. The patient was discharged from the ED with this recommendation and routine return precautions.

At the outpatient follow-up assessment with Neurosurgery 1 week later, the patient reported that her symptoms had worsened, with ascending leg weakness and paresthesia to her groin and anus. She noted that her ability to control her bowels and bladder was intact. Her examination showed diffusely decreased left lower extremity motor strength of 3/5 relative to 4/5 on the right. The remainder of the examination was unremarkable. There was no concern for ventriculoperitoneal shunt malfunction. Noncontrast cervical and thoracic spine MRIs were ordered for further evaluation, and the patient was encouraged to return to clinic when the imaging studies had been completed.

The patient returned to clinic 2 weeks later for reevaluation and imaging results review. The MRIs showed longitudinally extensive abnormal cord signal with enlargement, extending from the craniocervical junction to T1 with concern for longitudinally extensive transverse myelitis. The patient was advised to immediately present to the ED for admission to Hospital Medicine and further evaluation by the neurology and neurosurgery teams.

Within 24 hours of the patient's admission, a variety of tests were ordered to help elucidate her condition. Cerebrospinal fluid samples from her shunt showed lymphocytosis (89%, reference range, 40%-80%), the presence of red blood cells (16 cells/mm^3^, reference value, 0 cells/mm^3^), elevated oligoclonal bands (5 bands, reference value, <2 bands), and elevated kappa free light chains (0.1520 mg/dL, reference value, <0.1000 mg/dL). Serum and cerebrospinal fluid aquaporin-4 antibody testing and serum myelin oligodendrocyte glycoprotein testing were negative, effectively lowering seropositive neuromyelitis optica spectrum disorder on the list of differential diagnoses. All other laboratory findings, including serum and cerebrospinal fluid angiotensin-converting enzyme levels, were normal, and cultures yielded negative results.

The patient's abnormal cerebrospinal fluid findings raised concerns for multiple sclerosis, prompting an MRI demyelination protocol of the brain and cervicothoracic spine with and without contrast. MRI of the brain showed nonspecific, small white matter lesions of unclear etiology that were mild in degree, effectively lowering multiple sclerosis on the differential diagnosis list. MRI of the cervicothoracic spine demonstrated similar extensive cord edema from C2 through T6, with dorsal cord enhancement from C5 through T1. Axial postcontrast T1-weighted images revealed the characteristic trident sign that is highly suggestive of spinal cord sarcoidosis (Figure). At the time of the imaging studies, the patient displayed upper motor neuron signs: diffuse hyperreflexia and left lower extremity clonus. Her examination was consistent with the neuroimaging that showed an affected dorsolateral spinal cord with involvement of the dorsal columns and lateral corticospinal tract.

**Figure.**
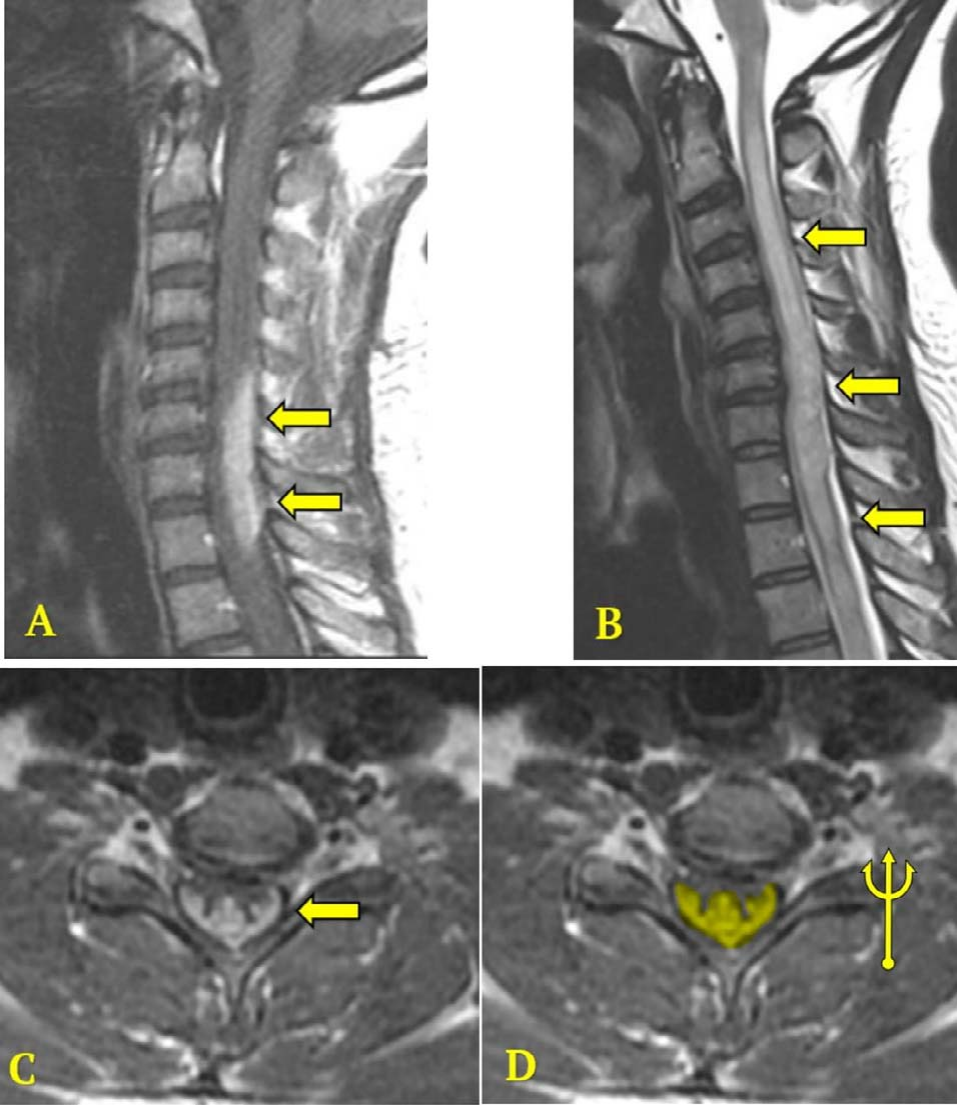
Magnetic resonance imaging of the spine. (A) Sagittal T1-weighted image with contrast shows extensive enhancement along the dorsal cord from C5 through T1 (arrows). (B) Sagittal T2-weighted image shows extensive signal abnormality throughout the visualized cervical and proximal thoracic cord (arrows). (C, D) Axial T1-weighted images with contrast show the characteristic trident sign (arrow and yellow highlight) corresponding to central cord enhancement with crescent-shaped dorsal subpial involvement.

Anterior posterior x-ray and noncontrast CT of the chest showed no evidence of cardiopulmonary disease or pulmonary sarcoidosis. Despite the initial suspicion for multiple sclerosis or neuromyelitis optica spectrum disorder, the patient's normal brain imaging, characteristic longitudinally extensive spinal cord lesion on spinal imaging, and negative aquaporin-4 and myelin oligodendrocyte glycoprotein tests were most consistent with spinal cord sarcoidosis.

With the presumptive diagnosis established, treatment was initiated with intravenous (IV) methylprednisolone sodium succinate at a dosage of 1,000 mg, administered over 30 minutes at a rate of 200 mL/h, repeated every 24 hours for 5 days. At this time, differential diagnoses being considered included seronegative neuromyelitis optica spectrum disorder, neoplasms, and other inflammatory and infectious processes. Workups for neoplasms, other inflammatory processes, and infection were negative.

During the patient's 5-day corticosteroid treatment course, the inpatient neurology team conducted 3 detailed sensorimotor neurologic examinations: the day after her admission, 3 days after her admission, and 4 days after her admission. The patient's first motor examination showed some weakness in the left upper extremity, with wrist flexion at 4+/5, finger abduction at 4-/5, and grip strength at 4/5 compared to 5/5 on the right. In the left lower extremity, hip flexion was 4-/5, knee flexion and extension were 4/5, and both dorsiflexion and plantarflexion were 4+/5 compared to 5/5 on the right. Reflexes were diffusely hyperactive at +3, with an equivocal plantar reflex and 2 beats of clonus in the left ankle. Sensory examination showed symmetric deficits in light touch, vibration, temperature, and pinprick in the distal bilateral lower extremities, with notably poor proprioception.

The patient's second sensorimotor examination was unchanged from the first, but her third motor examination showed some improvement in left lower extremity motor strength, with hip flexion, knee flexion and extension, and dorsiflexion and plantarflexion all at 4+/5. Reflexes had normalized to +2 bilaterally, and left ankle clonus was reduced to 1 beat. Sensory examination continued to show symmetric deficits in light touch, vibration, temperature, and pinprick in the distal bilateral lower extremities, as well as markedly poor proprioception.

Overall, the patient showed some improvement in left lower extremity strength, but ataxia and symmetric sensory deficits—particularly in proprioception—persisted despite treatment. During her 5-day inpatient stay, there was no pathologic confirmation of granulomatous disease or imaging indicative of pulmonary sarcoidosis. On the final day of IV corticosteroid treatment, the patient's condition was stable, but she still required assistive devices to ambulate. She was discharged with gabapentin 200 mg to be taken orally 3 times daily, home physical and occupational therapy orders, and the recommendation to follow up with outpatient neuroimmunology for further workup in 2 to 3 weeks.

Fifteen days after discharge, the patient visited the outpatient neuroimmunology clinic with complaints of continued sensory deficits and spastic gait. Motor examination showed wrist flexion and extension of 5/5 on the right and 4/5 on the left. Finger flexion was 5/5 on the right and 4+/5 on the left, while finger extension, abduction, and opposition were slightly weaker on the left at 4/5 compared to 5/5 on the right. Hip flexion was 4+/5 on the right and 4-/5 on the left, with knee flexion and extension of 5/5 on the right and 4/5 on the left. Plantar flexion was normal at 5/5 bilaterally, and dorsiflexion was 5/5 on the right and 4+/5 on the left. Sensory examination showed improved light touch sensitivity in the bilateral upper extremities that was greater than in the bilateral lower extremities, with numbness and reduced temperature sensation in the bilateral lower extremities. Vibration sense was considerably reduced in the bilateral lower extremities, absent up to the knees, and reduced in the fingers bilaterally. Proprioception was present in the bilateral upper extremities. Heel-to-shin testing was slightly ataxic on the left lower extremity. Gait was spastic with decreased cadence.

Despite the patient's improvements in left lower extremity motor strength, she continued to have a spastic gait with ataxia and sensory deficits in the bilateral lower extremities, particularly vibration sense and proprioception. She was encouraged to continue physical therapy, start a trial of baclofen 10 mg orally twice daily for symptom management, increase gabapentin to 300 mg orally 3 times daily, and undergo a same-day cervical MRI demyelination protocol to assess the radiologic response to her 5-day inpatient corticosteroid course. The MRI showed continued longitudinally extensive cord edema extending from C2 through the visualized upper thoracic spinal cord despite slight improvements in length that was most consistent with spinal cord sarcoidosis. After reviewing her imaging findings, the patient's neuroimmunologist recommended treatment in the hospital.

The patient self-admitted 8 days after her outpatient clinic visit and began another 5-day course of IV corticosteroids, along with 5 rounds of plasmapheresis using 2.5 L 5% albumin over 7 days. During the patient's inpatient stay, her gabapentin dose was increased to 400 mg orally 3 times daily. She tolerated plasmapheresis well, showing moderate improvements in bilateral lower extremities strength: right and left iliopsoas improved to 5/5 and 4+/5; right quadriceps improved to 5/5; and left hamstring, anterior tibial, and peroneal improved to 4-/5, 4/5, and 4/5, respectively. Her bilateral lower extremities sensory deficits remained unchanged. After completion of the final plasmapheresis treatment, the patient's condition was stable. She was discharged on gabapentin 400 mg orally 3 times daily, encouraged to continue home physical and occupational therapy, and recommended to follow-up with her neuroimmunologist in 2 weeks.

Fourteen days after discharge, the patient visited her neuroimmunologist with continued spastic gait and bilateral lower extremities sensory deficits. Motor examination showed improved bilateral hip flexion of 5/5, with continued vibration and proprioception sensory deficits in her bilateral lower extremities; left lower extremity ataxia on heel-to-shin testing; and spastic gait with decreased cadence. Given her mostly stable findings, a same-day whole-body positron emission tomography (PET) scan was ordered to investigate systemic sarcoidosis, and the patient was started on a high-dose oral steroid taper of prednisone 60 mg once daily for 1 month to bridge to infliximab immunotherapy. Whole-body PET results showed no abnormal tracer uptake to suggest systemic sarcoidosis, raising seronegative neuromyelitis optica spectrum disorder as a differential diagnosis.

After the patient's month-long high-dose steroid taper, she was started on IV infliximab at 5 mg/kg but developed nausea, vomiting, and fatigue that worsened with each consecutive induction dose. After 3 induction doses over 6 weeks, infliximab was held because of concerns for intolerance or an adverse reaction. Antibodies to infliximab were negative. Three months later, the patient had a virtual visit with her neuroimmunologist, who recommended two 1 g IV rituximab infusions every 2 weeks for 6 months for dual treatment of the patient's 2 most likely diagnoses: spinal cord sarcoidosis vs seronegative neuromyelitis optica spectrum disorder. She is still being seen for this complex condition and has not started treatment for rituximab because of insurance coverage issues.

## DISCUSSION

The diagnosis and management of spinal cord sarcoidosis are challenging because of the rarity of the condition and the overlap of clinical features with other neurologic conditions such as multiple sclerosis and neuromyelitis optica spectrum disorder. In our case, a middle-aged African American female presented with progressive neurologic deficits that initially raised suspicion for common neurologic disorders until imaging and laboratory evaluations pointed toward spinal cord sarcoidosis.

The diagnosis of spinal cord sarcoidosis is based on the clinical syndrome (myelopathy, neuropathic pain, paresthesia, bowel and bladder dysfunction, and Lhermitte sign), characteristic trident sign on axial MRI views, presence of noncaseating granulomatosis on histopathology, and exclusion of other causes. While no specific laboratory findings are associated with spinal cord sarcoidosis, in most cases, cerebrospinal fluid analysis shows pleocytosis and elevated protein levels.^14^

This case exemplifies the importance of considering spinal cord sarcoidosis as a differential diagnosis when patients present with longitudinally extensive transverse myelitis and display characteristic imaging findings. The trident sign observed on MRI in this patient was highly indicative of spinal cord sarcoidosis, aiding in the differentiation from other conditions such as multiple sclerosis and seropositive neuromyelitis optica spectrum disorder, which were initially suspected but subsequently ruled out based on serologic and imaging studies. Although the patient's PET scan did not show evidence of systemic sarcoidosis and raised the possibility of seronegative neuromyelitis optica spectrum disorder, the MRI findings were far more consistent with spinal cord sarcoidosis. The trident sign highlights the utility of advanced neuroimaging techniques in identifying spinal cord sarcoidosis, emphasizing the need for high clinical suspicion and thorough radiologic assessment in similar presentations.

The management of extrapulmonary sarcoidosis generally follows the same treatment algorithm as pulmonary sarcoidosis, which is largely based on small, uncontrolled trials and expert consensus because of the rarity of the disease and wide array of clinical presentations.^15^ Treatment generally requires the prompt initiation of corticosteroids at a starting dosage of 30 to 40 mg of oral prednisone per day, followed by a slow taper to a maintenance level of 7.5 to 10 mg over 6 months, although the regimen should be adjusted by severity of disease and response to treatment.^16^

The management of our patient demonstrates the complexities involved in treating spinal cord sarcoidosis. Initial high-dose corticosteroid therapy provided some improvement in motor strength, but sensory deficits and spastic gait persisted. Our patient's outcomes reflect the often partial and temporary response to steroids in spinal cord sarcoidosis, necessitating the consideration of additional immunosuppressive therapies. The use of plasmapheresis and subsequent trials of immunomodulatory treatments (eg, infliximab and rituximab) illustrate the iterative process of finding an effective therapeutic regimen tailored to the patient's response and tolerance.

This case also highlights the importance of a multidisciplinary approach in managing spinal cord sarcoidosis. Collaboration among the hospital medicine, rheumatology, neurology, neurosurgery, radiology, pathology, physical and occupational therapy, and pharmacy teams was crucial in providing comprehensive care. The involvement of a neuroimmunologist was particularly beneficial in guiding the diagnostic workup and treatment plan.

Furthermore, the patient's long-term management plan, which included a high-dose steroid taper and the need for rituximab infusions, reflects the need for ongoing immunosuppression to prevent relapse and manage chronic symptoms and aligns with current recommendations for the treatment of chronic and aggressive forms of extrapulmonary sarcoidosis that advocate for prolonged therapy with corticosteroids and immunomodulators for symptomatic improvement and sustained remission.^14,16-21^

## CONCLUSION

While current management guidelines for spinal cord sarcoidosis are based on limited evidence, further research is essential to establish definitive diagnostic and treatment protocols. The identification of the characteristic trident sign on axial MRI views may aid in distinguishing spinal cord sarcoidosis from similarly presenting conditions such as multiple sclerosis and neuromyelitis optica spectrum disorder. Early radiologic detection and diagnosis of this disease process could facilitate prompt initiation of appropriate treatment, potentially improving outcomes and reducing long-term neurologic deficits and sequelae for patients. Furthermore, a comprehensive multidisciplinary approach involving close collaboration among the patient and the hospital medicine, rheumatology, neurology, neurosurgery, radiology, pathology, physical and occupational therapy, and pharmacy teams is recommended for patient-centered care planning of this complex condition.

## Notes

*This article meets the Accreditation Council for Graduate Medical Education and the American Board of Medical Specialties Maintenance of Certification competencies for Patient Care and Medical Knowledge.*
